# Bone Morphogenetic Protein-9 Promotes Osteogenic Differentiation and Mineralization in Human Stem-Cell-Derived Spheroids

**DOI:** 10.3390/medicina59071315

**Published:** 2023-07-16

**Authors:** Sung-Bin Lee, Hyun-Jin Lee, Jun-Beom Park

**Affiliations:** 1 Dental Implantology, Graduate School of Clinical Dental Science, The Catholic University of Korea, Seoul 06591, Republic of Korea; sungbin25@naver.com; 2 Department of Periodontics, College of Medicine, The Catholic University of Korea, Seoul 06591, Republic of Korea; hyunjinlee0423@gmail.com

**Keywords:** bone morphogenetic proteins, cell differentiation, cell survival, gingiva, growth differentiation factor 2, stem cells

## Abstract

*Background and Objectives:* Alkaline phosphatase activity, mineralized matrix, and osteogenic-related gene expression have been shown to increase in response to bone morphogenetic protein-9 (BMP-9). In this study, spheroids derived from human gingival stem cells were used to determine the effects of BMP-9 on cell survival, osteogenesis, and mineralization. *Materials and Methods*: Human gingival stem cells were used to produce spheroids and then grown to concentrations of 0, 0.1, 1, 10, and 100 ng/mL with BMP-9. On days 1, 3, 5, and 7, morphological examination was carried out. A live/dead assay and Cell Counting Kit-8 was used to assess the vitality of cells. On days 7 and 14, alkaline phosphatase activity assays were carried out using a commercially available kit to examine the osteogenic differentiation of cell spheroids. Alizarin Red Staining was performed on the 7th and 14th days to evaluate mineralization, and *RUNX2* and *COL1A1* expression levels were evaluated on the 7th and 14th days using real-time polymerase chain reactions. *Results:* The BMP-9 added at the measured quantities did not appear to alter the shape of the well-formed spheroids produced by stem cells on day 1. In addition, treatment with BMP-9 at doses of 0, 0.1, 1, 10, or 100 ng/mL did not significantly alter cell diameter. Throughout the whole experimental process, viability was maintained. On day 14, the alkaline phosphatase activity in the groups dosed with 0.1, 1, 10, or 100 ng/mL was statistically higher than that in the unloaded control group (*p* < 0.05). According to qPCR data, the mRNA expression level of *RUNX2* with 1 ng/mL dosing was higher on day 7 compared to that of the unloaded control group (*p* < 0.05). *Conclusions:* These findings suggest that BMP-9 can be employed to stimulate early osteogenic differentiation in stem cell spheroids.

## 1. Introduction

BMPs, a family of signaling proteins that belong to the transforming growth factor superfamily, have enormous potential in the differentiation of mesenchymal stem cells (MSCs) [1]. BMPs have been demonstrated to cause MSCs to differentiate into bone and cartilage, in addition to their roles in adipogenesis [2,3], angiogenesis and lymphangiogenesis [4,5], boosting hepatocyte proliferation, various forms of metabolism, and many other vital activities [6]. Of the various BMPs, BMP-9 is one of those with the highest potential to induce the osteogenesis of MSCs in vitro and in vivo [7,8]. The potent osteogenic potential of BMP-9 is achieved by activating specific downstream mediators of osteogenic signaling and interacting with other signaling pathways [2,9,10,11]. BMP-9 is a powerful synergist for hematopoietic progenitor cell proliferation and colony formation, and it may play a role in the establishment and maintenance of neuronal cholinergic phenotypes in the central nervous system [12]. Recombinant human BMP-9 has been used in an animal model along with bioactive glass scaffolds to preserve the extraction site of teeth [13]. In recent studies, the strong osteogenic capacity of human dental MSCs stimulated with BMP-9 was demonstrated both in vivo and in vitro [14,15].

The microwell array has recently received a lot of interest due to the ease of cell seeding on this platform and the capacity of each well’s geometric homogeneity to generate uniform-sized spheroidal cell aggregates without the need for external stimulation [16,17]. Mesenchymal stem cell spheroids enhance anti-inflammatory effects, angiogenesis, immunomodulatory capabilities, stemness, and cell survival following transplantation [18,19]. The benefits of spheroid culture include increased viability, proliferation, and stemness by providing a similar physicochemical environment in vivo [20]. Spheroids are demonstrated to promote cell-to-cell communication [21]. The use of spheroids is reported to boost osteogenic differentiation when compared with a two-dimensional monolayer platform [22]. Growth factors or hydrogels can be applied to enhance the function of cell spheroids [23,24]. Gingiva-derived MSCs (GMSCs) show multipotent differentiation capabilities and powerful immunomodulatory effects, similar to MSCs taken from other tissues (such as bone marrow, adipose, or umbilical cord) [25,26,27,28]. An increasing body of research has shown that GMSCs have the potential to be a simple, expandable source of MSCs that can be accessed by minimally invasive surgical procedures [29,30]. Several studies have investigated the use of GMSCs for various applications, including bone regeneration and periodontal tissue engineering [31,32]. In this study, we used three-dimensional cultures of stem cell spheroids to investigate the effects of BMP-9 on the morphology of spheroids, cellular survival, and osteogenic differentiation. Given the existing studies involving BMP-9, we aimed to focus the present study on osteogenic differentiation.

## 2. Materials and Methods

### 2.1. The Current Study’s Design Involved GMSCs

**Figure 1:**
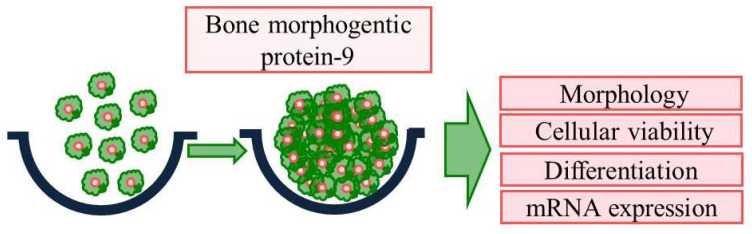
Diagram of the overall study flow.

Figure 1 presents an overview of the current investigation design. The current study protocol was assessed and authorized by Seoul St. Mary’s Hospital, College of Medicine, The Catholic University of Korea (KC22SISE0044; approval date 13 May 2022). Participants’ informed consent was acquired, and all experiments were conducted in accordance with the relevant principles and guidelines of the Declaration of Helsinki.

Previously established procedures were used to identify mesenchymal stem cells from gingiva with multipotent differentiation potential [33]. The gingiva tissues were de-epithelialized, cut into pieces, and digested using collagenase and dispase. The culture media changed every two days. In an incubator with 95% air and 5% CO_2_, the cells were cultivated at 37 °C.

### 2.2. Producing Stem Cell Spheroids

Stem cells were placed into concave silicon elastomer microwells (StemFIT 3D; MicroFIT, Seongnam-si, Gyeonggi-do, Republic of Korea) at a density of 1106 cells per well and grown in osteogenic medium. The final concentrations of recombinant human BMP-9 (3209-BP/CF; R&D Systems, Minneapolis, MN, USA) were 0, 0.1, 1, 10, and 100 ng/mL. An inverted microscope was used for morphological study on days 1, 3, 5, and 7 (CKX41SF; Olympus Corporation, Tokyo, Japan). The diameter of the spheres was measured by comparing the baseline lengths on days 1, 3, 5, and 7 in the image.

### 2.3. Evaluation of Cell Vitality

On days 1 and 7, a commercially available two-color assay based on esterase activity and plasma membrane integrity was used to determine cellular viability (Live/Dead Kit; Molecular Probes, Eugene, OR, USA) [34]. The spheroids were incubated with calcein acetoxymethyl ester and ethidium homodimer-1. In most eukaryotic cells, the cell-permeant dye calcein acetoxymethyl ester can be used to assess cell viability. After being hydrolyzed by intracellular esterases, the non-fluorescent calcein acetoxymethyl ester becomes green fluorescent calcein in living cells. Ethidium homodimer-1, a high-affinity nucleic acid stain that is faintly fluorescent until it binds to DNA and emits red fluorescence, is a cell-impermeant viability indicator. The spheroids were cultivated at room temperature for 60 min before being rinsed with growth media. Next, the stem cell spheroids were visualized with a fluorescence microscope (Axiovert 200; Carl Zeiss, Göttingen, Germany).

On days 1, 3, 5, and 7, a quantitative cellular viability test was carried out using the Cell Counting Kit-8, a water-soluble tetrazolium salt assay kit [35]. The cultures were treated with water-soluble tetrazolium salt and incubated for 1 h at 37 °C. The assay, which was used to assess living cells, is based on mitochondrial dehydrogenases’ ability to oxidize tetrazolium-8 into a formazan product.

### 2.4. Alkaline Phosphatase Activity Levels and Calcium Deposits

On days 7 and 14, alkaline phosphatase activity and an anthraquinone dye assay was performed to determine the level of osteogenesis [36]. On days 7 and 14, cell spheroids were formed on culture plates containing osteogenic medium. After adding cell lysates to an assay solution (K412-500; BioVision, Inc., Milpitas, CA, USA) containing a 5 mM p-nitrophenylphosphate substrate and incubating it at 4 °C for 30 min, the absorbance at 405 nm was evaluated [37]. In an alkaline buffer, alkaline phosphatase catalyzes the hydrolysis of phosphate esters to yield an organic radical and inorganic phosphate. The level and activity changes in alkaline phosphatase are related to the degree of osteogenic differentiation.

Alizarin Red S staining (Sigma-Aldrich, St. Louis, MO, USA) was used to determine calcium production. The bound dye was dissolved in a 10 mM sodium phosphate solution that included 10% cetylpyridinium chloride, and the concentration was determined spectrophotometrically at 560 nm [38,39].

### 2.5. Real-Time qPCR Measurement of mRNA for RUNX2 and COL1A1

Total RNA extraction was carried out according to the manufacturer’s instructions using a commercial kit (Thermo Fisher Scientific, Inc., Waltham, MA, USA) [40]. RNA quality was evaluated using a spectrophotometer (ND-2000; Thermo Fisher Scientific, Inc.) and an absorbance ratio of 260 to 280 nm. The reverse transcriptase (SuperScript II; Invitrogen, Carlsbad, CA, USA) employed RNA as a template for reverse transcription.

On days 7 and 14, mRNA expression was determined using qPCR. To create the sense and antisense PCR primers, we used GenBank. The primer sequences are detailed as follows: for *RUNX2*, we used the notation NG/ML 001015051.3; for *COL1A1*, we used the notation NG/ML 000088.4; and for β-actin, we used the notation NG/ML 001101; forward (5’-AATGCTTCTAGGCGGACTATGA) and reverse (5’-TTTCTGCGCAAGTTAGGTT). Real-time PCR was performed on a thermocycler (StepOnePlus; Applied Biosystems, Waltham, MA, USA) using the SYBR Green PCR Kit (Applied Biosystems) in accordance with the manufacturer’s instructions [41,42].

### 2.6. Statistic Assessment

For each variable, the mean and standard deviation were computed. The program (SPSS 12 for Windows, SPSS Inc., Chicago, IL, USA) was used to evaluate normality and variance equality tests. The groups were compared using one-way ANOVA and Tukey’s post hoc test. For each analysis, three experimental duplicates were examined. The significance level was set at 0.05.

## 3. Results

### 3.1. Human GMSC Spheroids

**Figure 2:**
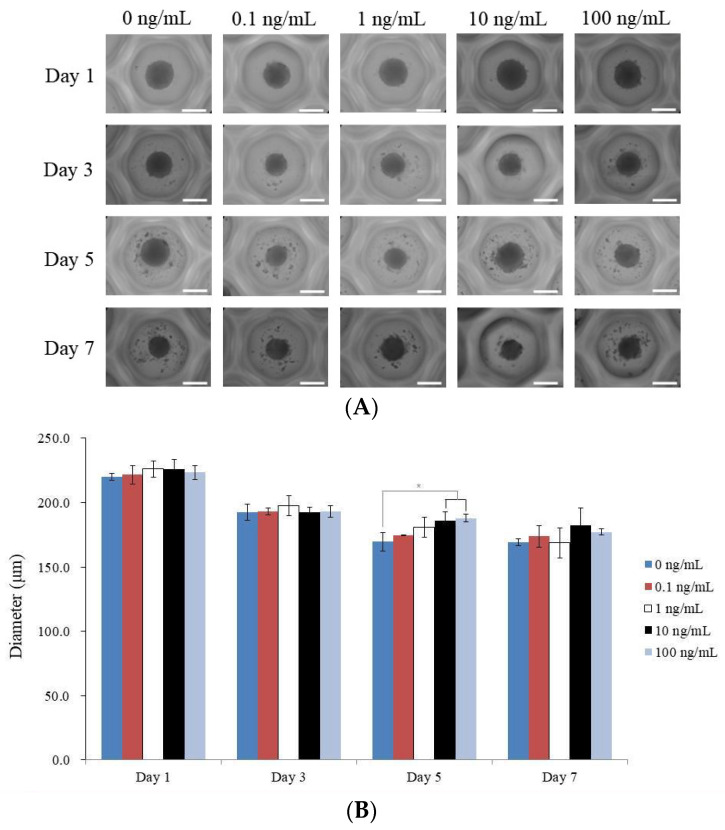
Evaluation of the morphology of stem cell spheroids. (**A**) BMP-9 concentration-dependent morphologies of stem cell spheroids on days 1, 3, 5, and 7. The length of the scale bar is 200 μm. (**B**) Stem cell spheroids’ dimensions. * *p* < 0.05 compared to 0 ng/mL group by time zone.

This section covers an experiment where BMP-9 was applied to stem cell spheroids at various concentrations. Figure 2A illustrates the shape of the spheroids at various time points (days 1, 3, 5, and 7). Figure 2B shows the diameter of the spheroids at day 1 for each BMP-9 concentration group, as well as the trend in size change over the course of the seven-day incubation period. The spheroids were all initially circular and exhibited no appreciable shape changes during the experiment. Each BMP-9 concentration group had spheroids that ranged in diameter from 220.0 µm (for the group that had no BMP-9) to 226.2 µm (for the groups that had 10 and 100 ng/mL of BMP-9) on day 1. On day 1, the diameters of the various BMP-9 concentration groups did not differ significantly. The diameter of the spheroid shrank gradually throughout the course of the seven-day incubation period. We did not specify precise values for every day or concentration group, but this decline was similar in all groups.

### 3.2. Determining the Vitality of Cell Spheroids Qualitatively and Quantitative Values for Cell Spheroid Viability

**Figure 3:**
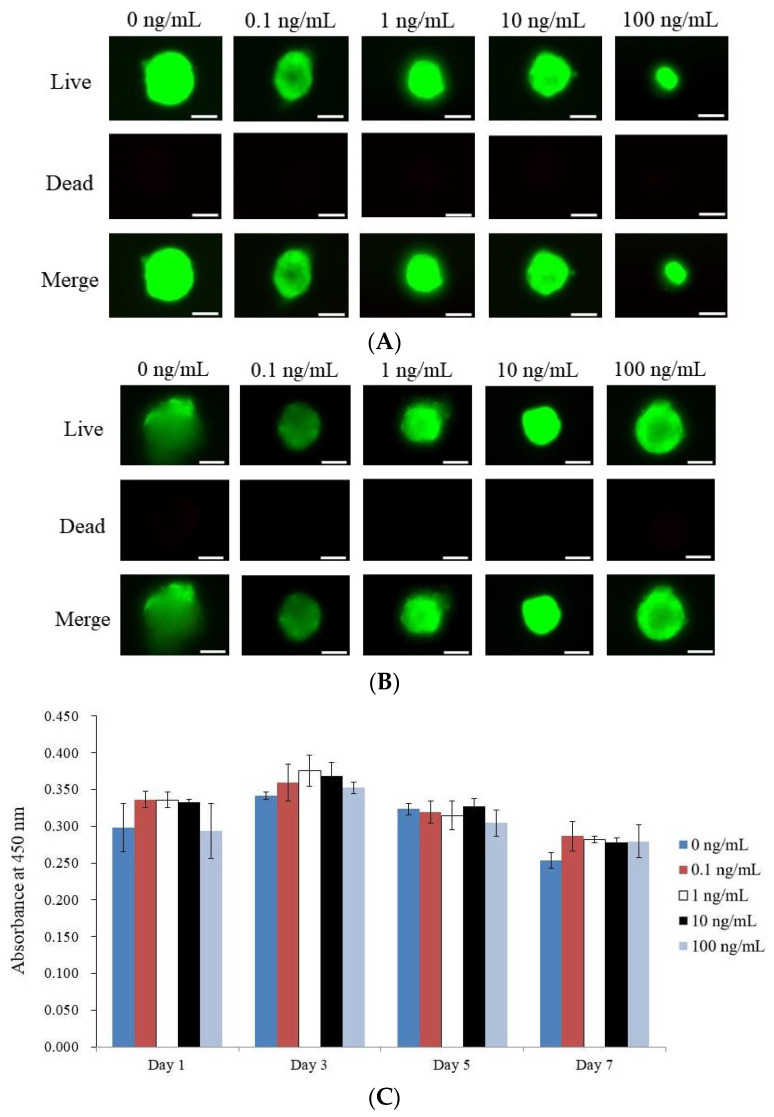
Cell viability measurements were collected on days 1 and 7. (**A**) Stem cell spheroids on day 1 were captured in optical, live, dead, and merged cell pictures. (**B**) On day 7, living, dead, and merged cell images of stem cell spheroids were obtained. The length of the scale bar represents 50 μm. (**C**) Cell viability was measured on days 1, 3, 5, and 7 using Cell Counting Kit-8.

Here, the outcomes of a Live/Dead Kit carried out on stem cell spheroids exposed to various amounts of BMP-9 on days 1 and 7 are discussed. Using green fluorescence to indicate live cells and red fluorescence to indicate dead cells, the experiment assessed the viability of the spheroids. The bulk of the stem cells fluoresced bright green on day 1, indicating that they were alive (Figure 3A). On day 7, there was no noticeable decrease in green fluorescence, demonstrating that the spheroids were viable during the whole seven-day incubation period (Figure 3B).

The quantitative cellular vitality of the stem cell spheroids on days 1, 3, 5, and 7 is shown in Figure 3C. On day 1, the absorbance values for the various BMP-9 concentration groups were not statistically different from one another (*p* > 0.05), demonstrating that BMP-9 treatment had no effect on the initial viability of the cells. The stem cell spheroid viability did not significantly change over the seven-day incubation period.

### 3.3. Evaluating Alkaline Phosphatase Activity and Alizarin Red S Staining

**Figure 4:**
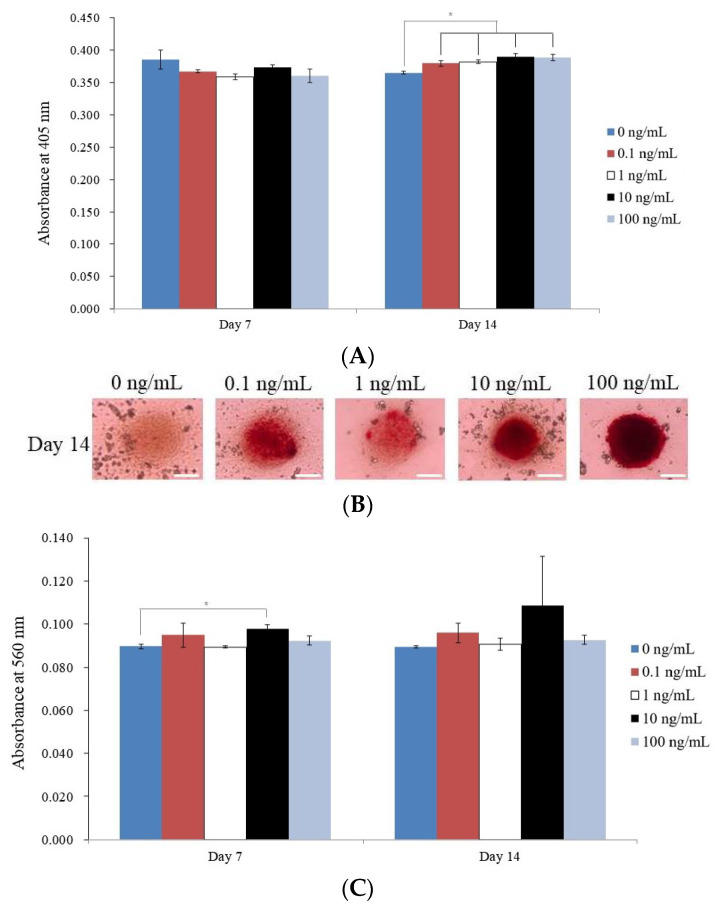
Differentiation in osteogenesis. (**A**) Graphs are used to display the results of the alkaline phosphatase activity tests conducted on days 7 and 14. * On day 14, *p* < 0.05 compared to the time-matched 0 ng/mL group. (**B**) On day 14, the outcomes and results of Alizarin red S staining. The scale bar denotes 100 μm. (**C**) Alizarin red S staining measurement. * On day 7, *p* < 0.05 compared to 0 ng/mL group by time zone.

This section details the outcomes of various studies on stem cell spheroids treated with varying doses of BMP-9. The absorbance value of each BMP-9 concentration group was examined on day 14, and there was a significant difference between the groups (*p <* 0.05). The untreated control group had the lowest absorbance value (0.365 ± 0.002), whereas the 10 ng/mL group had the highest absorbance value (0.390 ± 0.006). On day 14, alkaline phosphatase activity was also assessed, and the results revealed a substantial increase in activity compared to the control in the 0.1, 1, 10, and 100 ng/mL BMP-9 concentration groups (*p* < 0.05). The data for alkaline phosphatase activity on day 14 are shown in Figure 4A.

On day 14, calcium deposits were seen in each BMP-9 concentration group, as depicted in Figure 4B. For every BMP-9 concentration group, the absorbance values for Alizarin Red S staining were also determined on days 7 and 14. On day 7, the 10 ng/mL group had the highest absorbance value (0.098 ± 0.002), as it did on day 14 (0.109 ± 0.023). Treatment with BMP-9 significantly increased Alizarin Red S staining values compared to those of the control group, with the 10 ng/mL group achieving the highest value on day 7 (*p <* 0.05). The absorbance values for Alizarin Red S staining on days 7 and 14 are displayed in Figure 4C.

### 3.4. Analysis of RUNX2 and COL1A1 by qPCR

**Figure 5:**
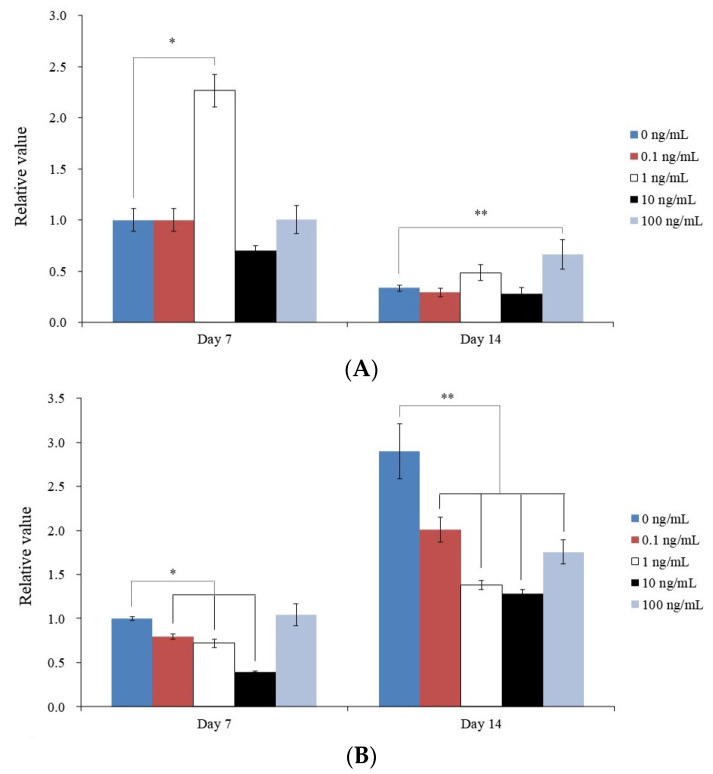
Expression of mRNA in a quantified manner. (**A**) Measurement of *RUNX2* mRNA expression. * *p* < 0.05 compared to 0 ng/mL group by time zone. ** *p* < 0.05 compared to the 0 ng/mL group on day 14. (**B**) *COL1A1* mRNA expression on days 7 and 14. * *p* < 0.05 compared to the 0 ng/mL group on day 7. ** *p* < 0.05 compared to 0 ng/mL group by time zone.

This section details the findings of a qPCR investigation of the levels of *RUNX2* and *COL1A1* mRNA on days 7 and 14 at various concentrations of BMP-9 (0, 0.1, 1, 10, and 100 ng/mL). *RUNX2* mRNA expression increased following the injection of 100 ng/mL BMP-9 (*p <* 0.05) (Figure 5A). Meanwhile, the expression of *COL1A1* mRNA decreased following the addition of BMP-9 (Figure 5B). These findings imply that *COL1A1* and *RUNX2* mRNA expression can both be modified by BMP-9.

## 4. Discussion

This study examined how human MSC spheroids respond to BMP-9 in terms of cell viability, osteogenic differentiation, and mineralization. Alkaline phosphatase activity and *RUNX2* mRNA level, which were measured by real-time qPCR, were used to identify differentiation into an osteogenic lineage. We found that the application of BMP-9 increased alkaline phosphatase activity, Alizarin Red S staining, and the expression of *RUNX2,* without affecting cellular viability. However, stem cell spheroids treated with BMP-9 did not display an increase in *COL1A1* mRNA expression, in agreement with a previous study [43]. This study demonstrated alkaline phosphatase activity and the mineralization of human MSCs induced by BMP-9 in two-dimensional cultures; however, three-dimensional scaffolds did not show any appreciable variations in collagen I expression, osteopontin expression, or mineralization. Instead, BMP-9 increased the expression of the cartilage oligomeric proteins Sox9, aggrecan, and collagen II [43]. 

Rat dental follicle stem cells transduced by BMP-9 displayed enhanced alkaline phosphatase activity, mineralization, and expression of bone-related genes in a previous three-dimensional cell culture investigation (*DLX5*, *OPN*, *Osx*, and *RUNX2*) [44]. The BMP-9 dosage varied in various experimental contexts [13,45,46,47]. A low dose of 1 ng/mL of BMP-9 was used to treat the cells. In prior work, cells were exposed to a low concentration of BMP-9 (1 ng/mL) and high concentration of 5 ng/mL [45]. Then, using four groups and recombinant human BMP-9 at low (10 ng/mL) and high (100 ng/mL) concentrations on an absorbable collagen sponge, the cellular effects of stromal cell line preosteoblasts were examined [46]. The findings show that, in contrast with recombinant human bone morphogenetic protein-2 at concentrations as much as 10 times lower, recombinant human BMP-9 had a considerable influence on osteoblast development when paired with an absorbable collagen sponge [46]. In a different study, 5 × 10^3^ synovial MSCs were cultivated with BMP-9 at concentrations between 10^−7^ and 10^−3^ mol/L [47]. Recombinant human BMP-9 is incubated at a concentration of 100 ng/mL onto bioceramic material after a soaking period in a shaking incubator at 37 °C [7]. The calvarial bone deficiencies were filled with a 3 mm diameter circle of collagen membrane and 500 ng of recombinant human BMP-9 [48]. In our study, doses of 0, 0.1, 1, 10, and 100 ng/mL were used, and it was difficult to determine the optimal concentration for osteogenic differentiation. It would be meaningful to conduct further studies using higher concentrations of BMP-9.

The following can be used to describe the effects of BMP-9 [47,49,50]. By triggering the JNK/Smad2/3 signaling pathway, BMP-9 stimulated the osteogenic development of synovial MSCs [47]. According to reports, BMP/Smad and PI3K/AKT/m-TOR signaling pathways are important in controlling the coordinated effects of osteogenic and angiogenic pathways [49]. A small amount of transforming growth factor-1 could counteract the hypertrophic impact of BMP-9 [50]. It was shown that SIRT1-Smad7 interaction boosted Smad7 degradation and elevated Smad1/5/8 phosphorylation [51].

Ex vivo gene therapy using human MSCs and bone morphogenetic protein genes provides a local supply of precursor cells, as well as a hyperphysiological capacity of bone-inducing molecules, which can promote bone formation in patients with aging, osteoporosis, metastatic bone disease, iatogenicity depletion, insufficient human MSC populations, or other metabolic disorders [52,53,54]. The enormous amounts of ectopic bone shown in computed tomographic tests and histological examination at the injection sites for human MSCs transduced with BMP-9 resulted in effective spinal fusion with no evidence of nerve root compression or local or systemic damage [52].

This study is unusual in that it investigates how BMP-9 influences cell survival, osteogenic differentiation, and the mineralization of human gingiva stem cell spheroids. This study focuses on the impact of BMP-9 on human GMSCs, a promising source of stem cells for regenerative medicine. Furthermore, the current study looks into different BMP-9 concentrations to determine the optimal dosage for supporting osteogenic differentiation. Overall, this study adds to our understanding of the use of BMP-9 to promote the osteogenesis differentiation of stem cell globules that may affect future treatments, such as regenerative medicine. For individuals with insufficient stem cell populations to encourage bone growth, ex vivo gene therapy employing human MSCs and bone morphogenetic protein genes may be a viable option.

The study did have limitations. First, the results of this study may not accurately reflect the circumstances in vivo because it was carried out in vitro. Spheroids are a good model for in vitro research, but they do not have the intricate three-dimensional architecture of human body tissues and organs [55]. Five concentrations of BMP-9 were used in the study to examine its effects, although they might not accurately represent in vivo situations. Only human-gingiva-derived stem cells were used in this study; other cell types may react differently to BMP-9 [56]. Moreover, the duration of this trial was only 14 days; thus, it is likely that longer exposure to BMP-9 could result in different outcomes [57]. Larger sample sizes could be required to confirm the results because the study sample size was rather small.

More research is needed into the methods by which BMP-9 promotes osteogenic differentiation in stem cell spheroids. This would entail looking into the signaling pathways at play and identifying other elements that might amplify the effects of BMP-9. The investigation might also be broadened to consider how BMP-9 might be used in tissue engineering or bone regeneration in the clinic. The three-dimensional cultures of spheroids composed of mesenchymal stem cells may require future standardization and an in-depth investigation of their biophysical properties [58]. This could entail investigating the safety and effectiveness of BMP-9 in human clinical trials, as well as studying the effects of BMP-9 on bigger tissue constructs or in animal models. Overall, the results of this study lay the groundwork for future investigations into the subject of regenerative medicine and have the potential to produce fresh approaches to bone and tissue restoration.

## 5. Conclusions

The current research is important for bone formation and regenerative medicine because it sheds light on the possibility of using BMP-9 to promote osteogenesis differentiation in stem cells produced from human gums. The facile accessibility and number of gingiva-derived stem cells demonstrate that they are a desirable source of MSCs for cell therapy. The results of this study show the possibility of enhancing the osteogenic differentiation of human-gingiva-derived stem cells in vitro utilizing BMP-9, which may have significant uses for bone regeneration and repair in clinical situations. However, more studies and clinical trials are required to assess the security and effectiveness of human-gingiva-derived stem cells and BMP-9 in cell therapy. These results led us to the conclusion that BMP-9 can be used to promote early osteogenic differentiation in stem cell spheroids.

## Data Availability

All data analyzed during this study are included in this published article.
